# Supplementary material for: Accelerating the characterization of dynamic DNA origami devices with deep neural networks
Source: Sci Rep. 2023 Sep 14;13:15196. doi: 10.1038/s41598-023-41459-w (PMC10502017; doi:10.1038/s41598-023-41459-w)
Supplement: Supplementary file 1 — Supplementary Information. [file 41598_2023_41459_MOESM1_ESM.pdf]

Accelerating the Characterization of Dynamic DNA Origami Devices with Deep Neural Networks

Yuchen Wang[1]*, Xin Jin[2], Carlos Castro[1]*

Department of Mechanical and Aerospace Engineering, The Ohio State University, Columbus, OH 43210, USA

Department of Computer Science and Engineering, The Ohio State University, Columbus, OH 43210, USA

Supplemental Information

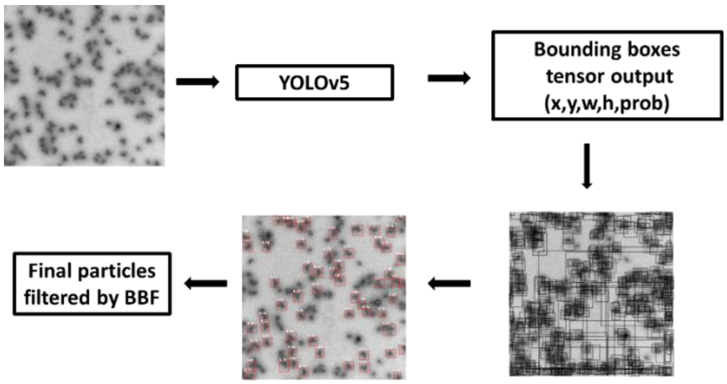

Figure S1 Generalized 'particle detection problem' input-output inference workflow. The images are processed by YOLOv5 after multi-stage convolutional operations. The output of network are a set of grids with each box has x, y, w, h, probability, respectively. The data on the grid was converted into bounding box. The probability value was used as threshold to balance the network precision and recall. See https://github.com/ultralytics/yolov5 for details. Finally, the BBF was applied to further improve the network performance.

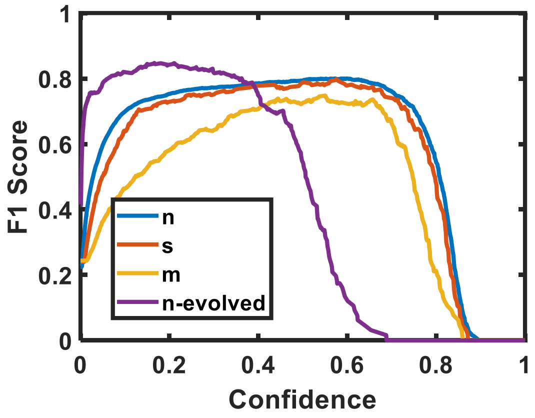

Figure S2 The performance comparison between several models provided by YOLOv5 with different complexities. n: nano, s: small, m: medium. Evolved represents a model trained by using an optimized hyperparameter from the genetic algorithm. Training size =10 images.

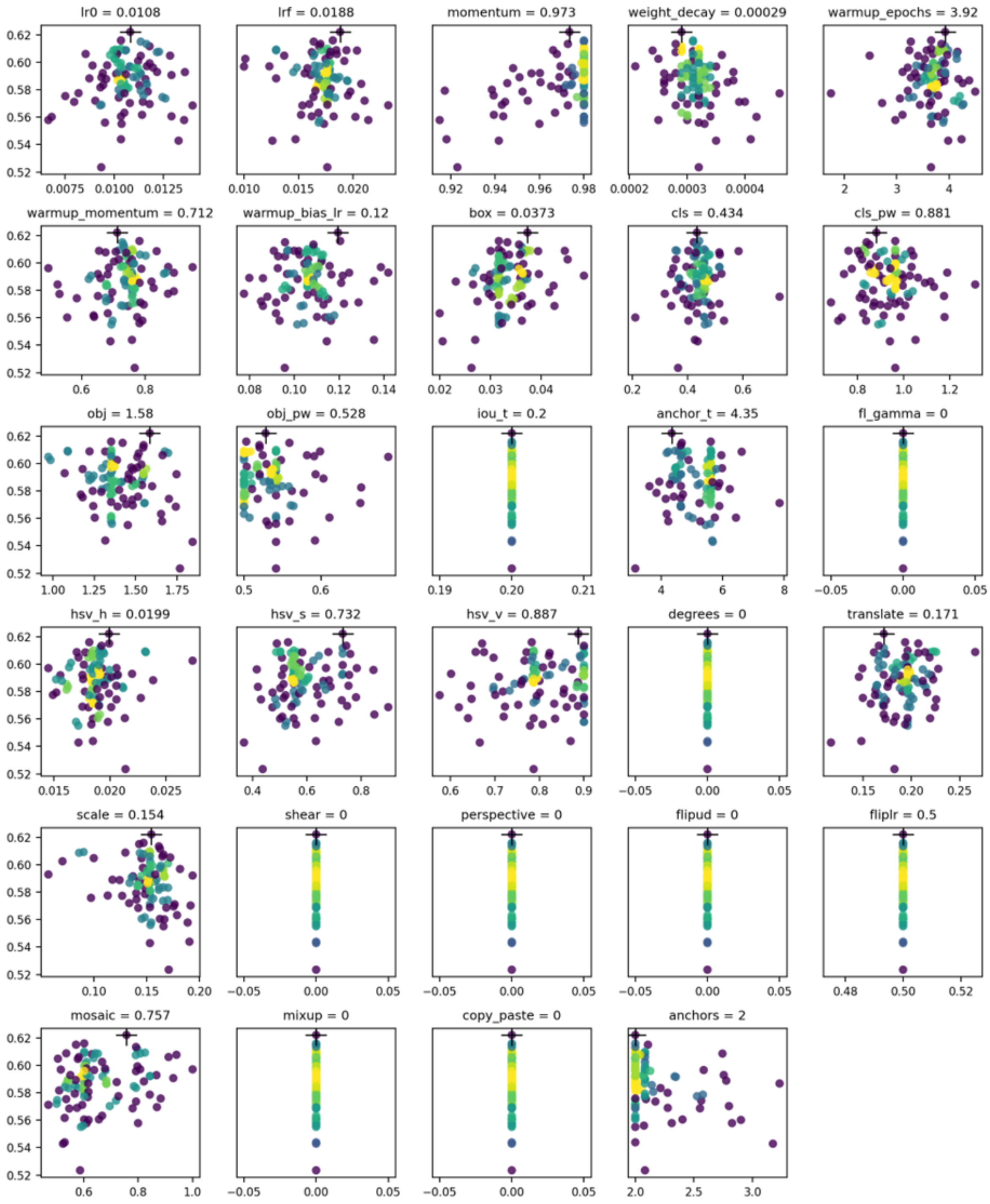

Figure S3 Optimization of hyperparameters matrix by genetic algorithm. The X axis is the hyperparameter value and the Y axis is the fitness value. The fitness function was defined as f=0.1*(mAP@0.5) +0.9*(mAP@0.5:0.95). Color represents dots density. The final hyperparameters were selected with the highest fitness after 300 generations with 500 epochs of each.

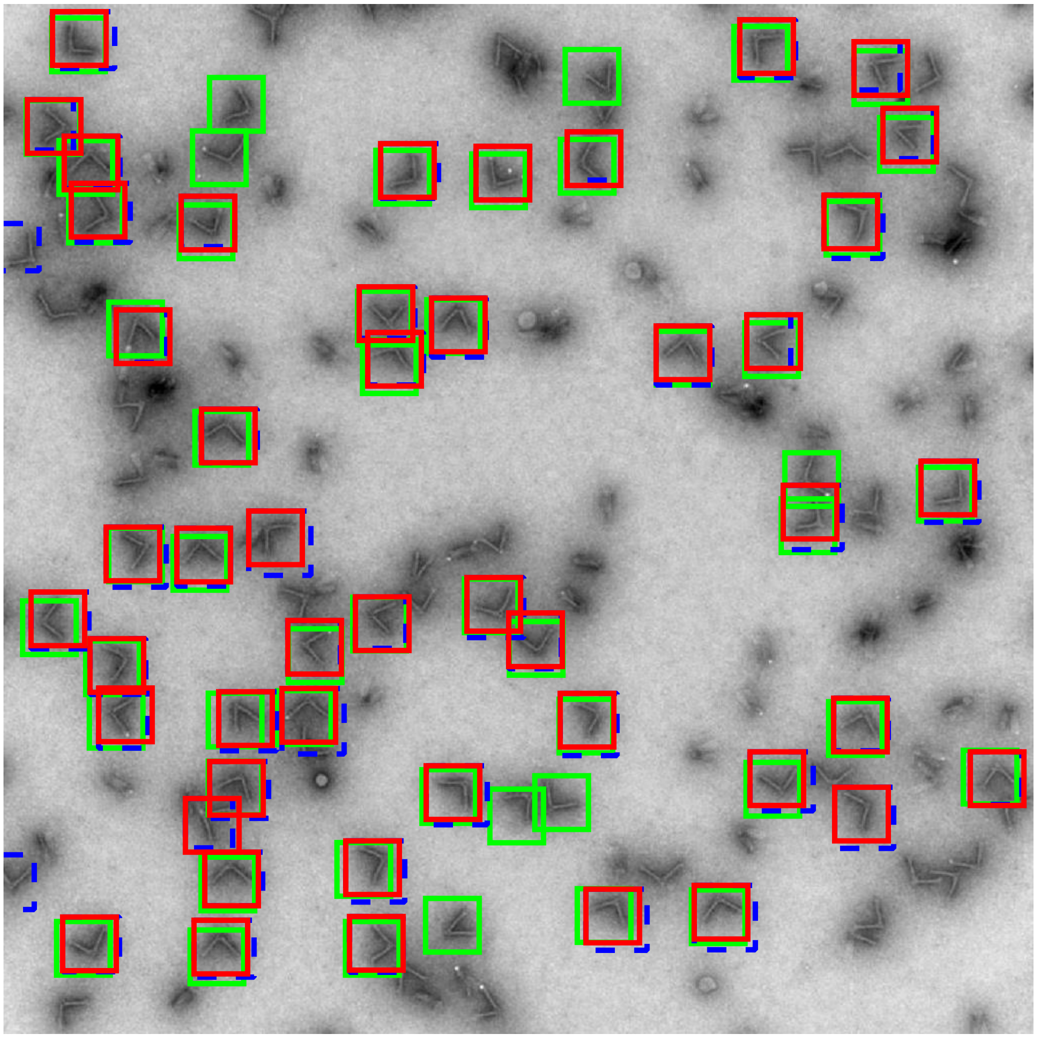

Figure S4 Comparison between ground truth (green), prediction for YOLOv5 (blue), and prediction after BBF (red). The blue-red offset effect is due to the resize of bounding box from predicted weigh and height into 50x50 pixel.

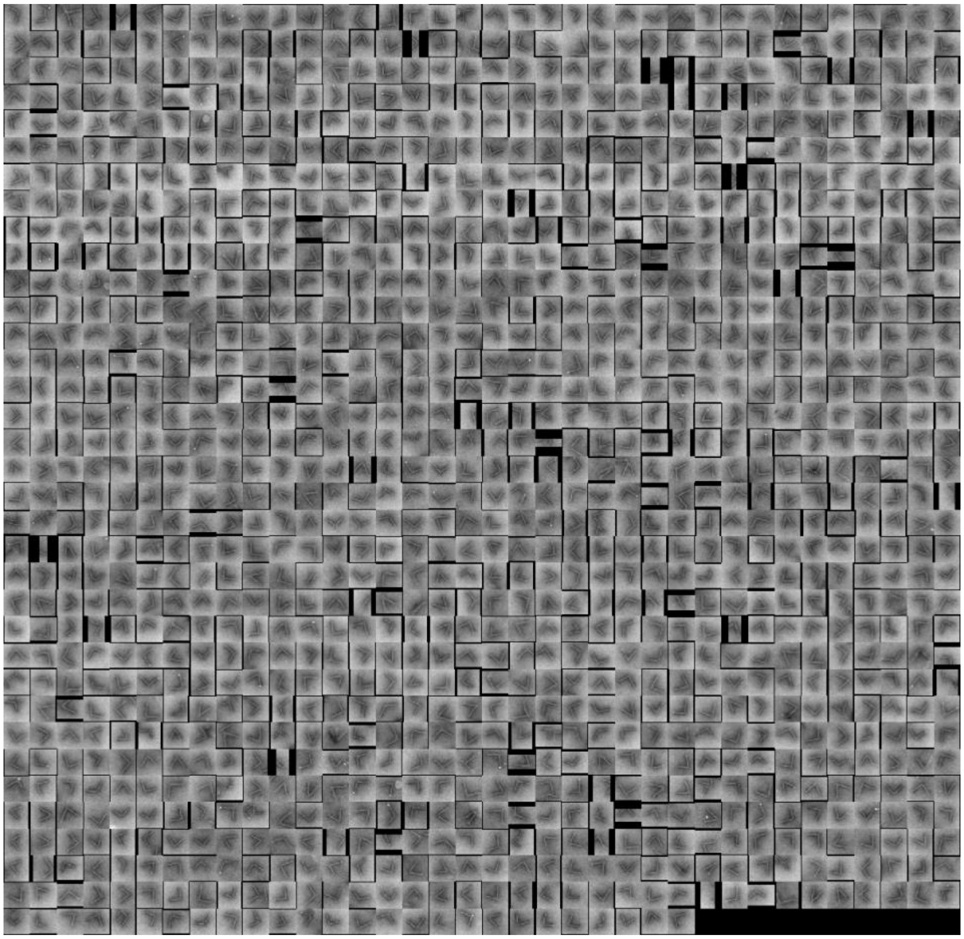

Figure S5 A gallery collection of predicted hinges from YOLOv5.

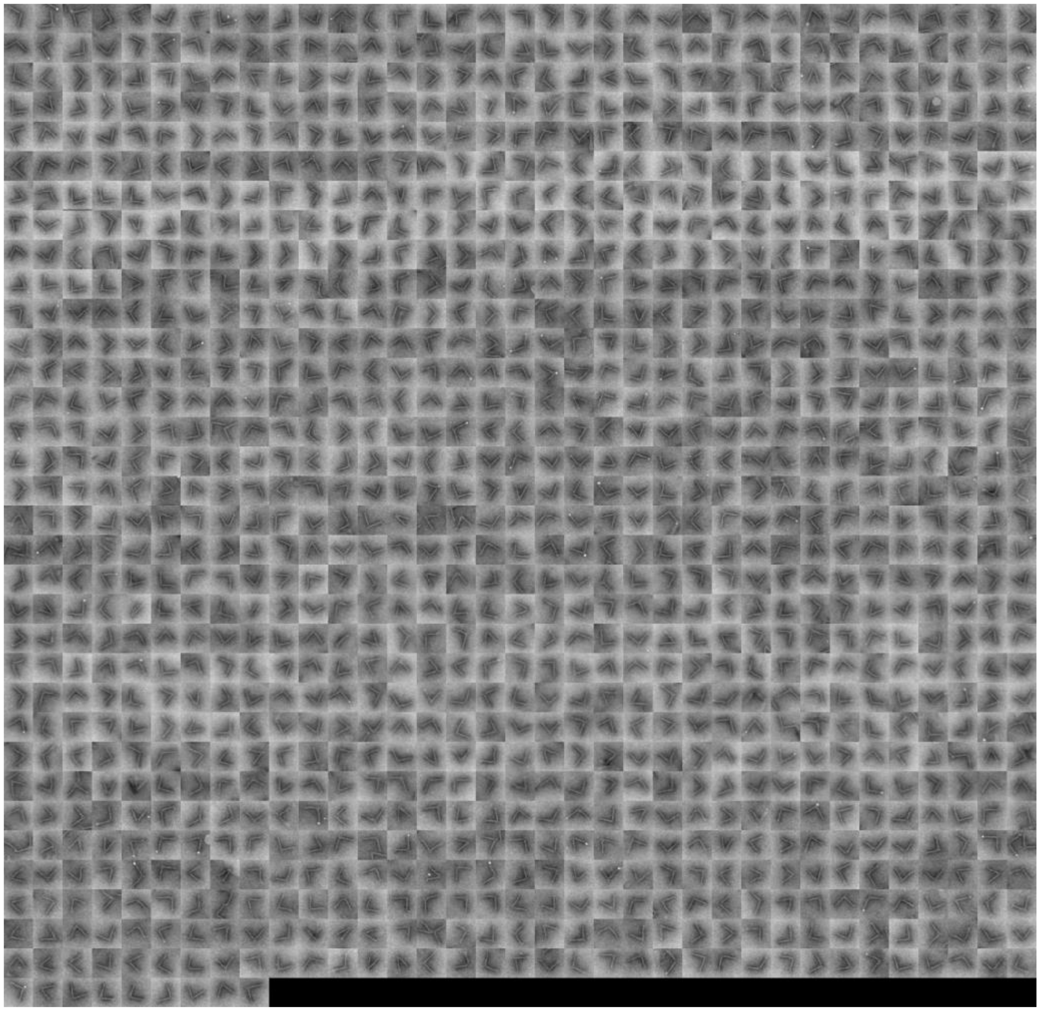

Figure S6 A gallery collection of predicted from YOLOv5 after BBF.

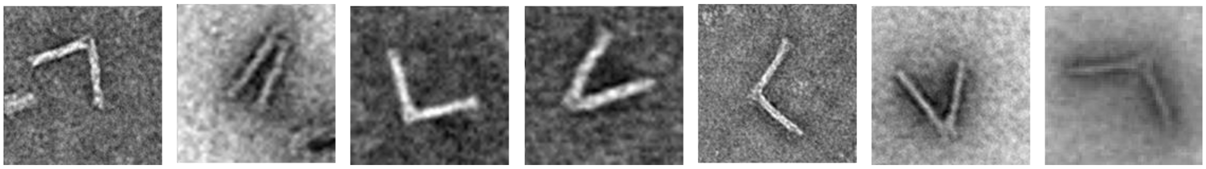

Figure S7 Heavy metal Uranyl formate staining process to TEM grid gives random image contrast behavior.

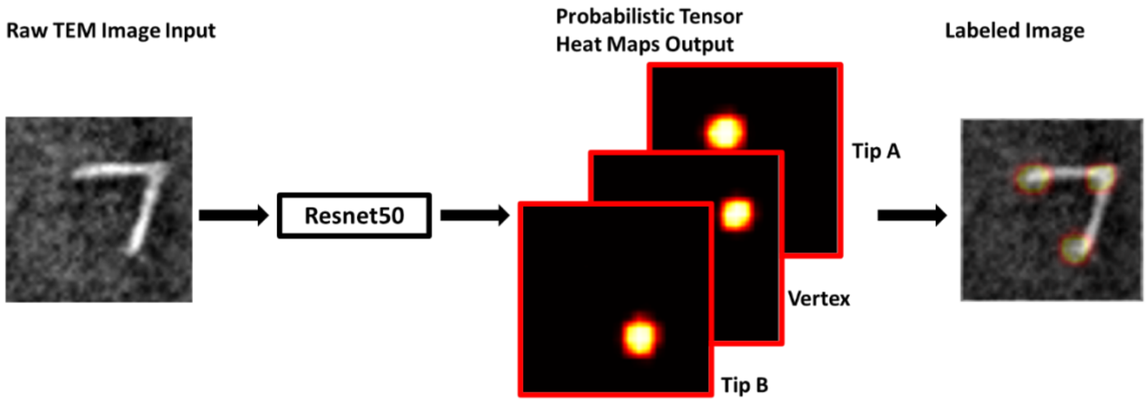

Figure S8 Forward evaluation process to determine the pose in Resnet50.

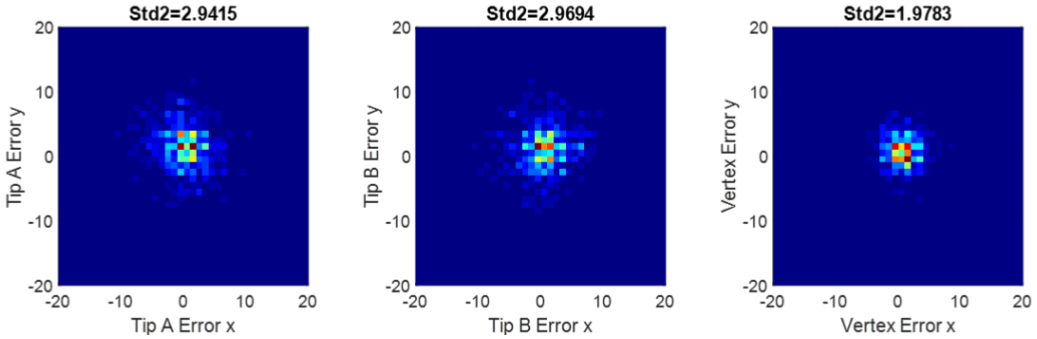

Figure S9 Comparison of between two annotators in a sub-dataset (616 particles). The Tip A and Tip B will be flipped when the following condition satisfied: dist(A,A')+dist(B,B')>dist(A,B')+dist(A',B), where 'dist' represents the Ellucian distance between two points, prime represents the other annotator.

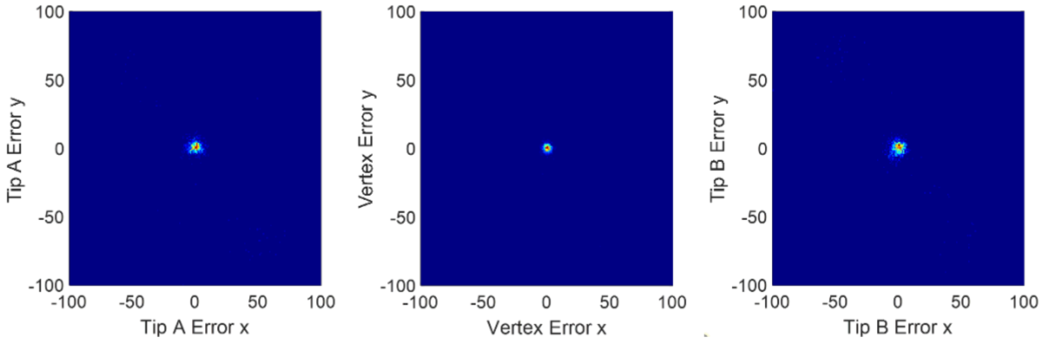

Figure S10 Hinge pose error shown with larger axis limits.

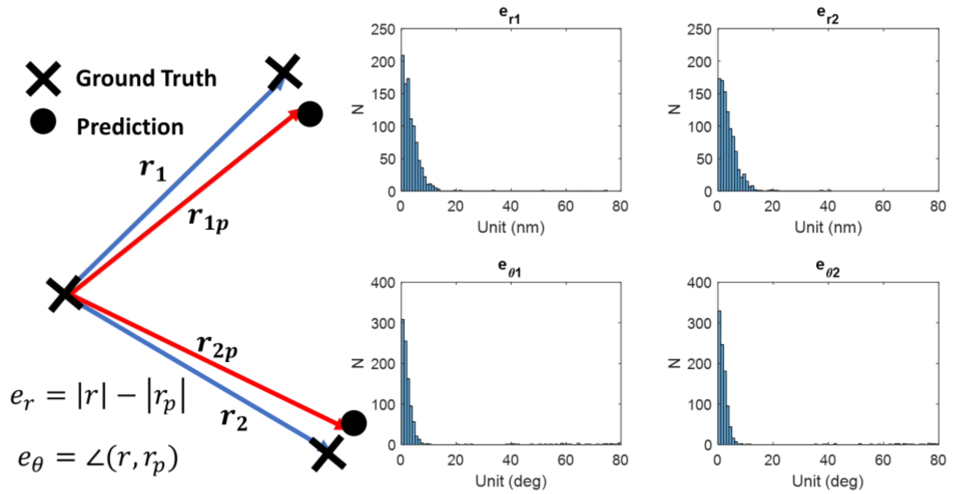

Figure S11 Hinge pose estimation error in terms of radial length and theta. Mean values of error r1, r2, theta1, theta2 are 3.44 nm,3.76 nm, 6.2 deg, 6.0 deg, respectively.

Estimation of image analysis workload before and after applying deep learning pipeline – A case study for the hinge structure

Workload after applying deep learning pipeline:

Workload to prepare Raw TEM Image Training Dataset

= Bounding boxing process per hinge (5 seconds) × Number of TEM training images (10 images) × Number of useful DNA origami structures per TEM images (50/image)

= 2500 seconds

Workload to prepare Image Particle Training Dataset

= Pose annotation per hinge (10 seconds) x Number of hinge training particles (700 particles)

= 7000 seconds

The total workload for the prepare deep learning pipeline is 9500 seconds or 2.6 hours. Additionally, it is worth mentioning that the time for the inference process from neural networks is negligible.

Workload before applying deep learning pipeline:

Note that the annotation work is independent for different hinge conditions, thus every single experimental modification needs a complete image analysis process.

In practice, different free energy landscapes are required to fulfill the force spectrometer functions. As an example, we reported 17 experimental conditions in this prior study [1], and we had tested more than 20 other conditions during the design and testing phase of that prior study. This gives us a total of greater than 37 different conditions that we had performed manual angle measurements on. We apply a similar estimation for each condition and assume we need at least 300 particles for each condition to provide a good statistical sampling of the free energy landscape.

Workload to prepare Raw TEM Image Dataset

= Bounding boxing process per hinge (5 seconds) × Number of particles bounding box needed per condition (300 particles)

= 1500 seconds

Workload to prepare Image Particle Dataset

= Pose annotation per hinge (10 seconds) x Number of hinge particles per condition (300 particles)

= 3000 seconds

The total workload for the prepare deep learning pipeline is 4500 seconds or 1.25 hours.

Therefore, it would not be worth using a deep learning pipeline when there is only one condition to be analyzed. However, the total estimated workload to manually analyze all 37 conditions would be 46.25 hours, which compared to the estimated workload of 2.6 hours with the deep learning pipeline clearly illustrates the drastic time savings for cases where multiple conditions will be evaluated. Even though there are 1-2 hours required for training time, and code set-up time, the deep learning pipeline is a much more efficient approach.

Reference:

[1] Y. Wang *et al.*, “A nanoscale DNA force spectrometer capable of applying tension and compression on biomolecules,” *Nucleic Acids Research*, vol. 49, no. 15, pp. 8987–8999, 2021, doi: 10.1093/nar/gkab656.
